# A randomised controlled study of high intensity exercise as a dishabituating stimulus to improve hypoglycaemia awareness in people with type 1 diabetes: a proof-of-concept study

**DOI:** 10.1007/s00125-019-05076-5

**Published:** 2020-01-15

**Authors:** Catriona M. Farrell, Alison D. McNeilly, Paul Fournier, Timothy Jones, Simona M. Hapca, Daniel West, Rory J. McCrimmon

**Affiliations:** 1 Division of Systems Medicine, School of Medicine, University of Dundee, Dundee, DD19SY UK; 2 Faculty of Science, School of Human Science, The University of Western Australia, Perth, Australia; 3 Faculty of Health and Medical Sciences, Paediatrics, The University of Western Australia, Perth, Australia; 4 Computing Science and Mathematics, Faculty of Natural Sciences, University of Stirling, Stirling, UK; 5 Institute of Cellular Medicine, Faculty of Medical Science, Newcastle University, Newcastle, UK

**Keywords:** Behaviour, Counterregulation, Diabetes, Exercise, Habituation, Hypoglycaemia, Impaired awareness

## Abstract

**Aims/hypothesis:**

Approximately 25% of people with type 1 diabetes have suppressed counterregulatory hormonal and symptomatic responses to insulin-induced hypoglycaemia, which renders them at increased risk of severe, disabling hypoglycaemia. This is called impaired awareness of hypoglycaemia (IAH), the cause of which is unknown. We recently proposed that IAH develops through habituation, a form of adaptive memory to preceding hypoglycaemia. Consistent with this hypothesis, we demonstrated restoration of defective counterregulatory hormonal responses to hypoglycaemia (referred to as dishabituation) in a rodent model of IAH following introduction of a novel stress stimulus (high intensity training [HIT]). In this proof-of-concept study we sought to further test this hypothesis by examining whether a single episode of HIT would amplify counterregulatory responses to subsequent hypoglycaemia in people with type 1 diabetes who had IAH (assessed by Gold score ≥4, modified Clarke score ≥4 or Dose Adjustment For Normal Eating (DAFNE) hypoglycaemia awareness rating 2 or 3). The primary outcome was the difference in adrenaline response to hypoglycaemia following both a single episode of HIT and rest.

**Methods:**

In this randomised, crossover study 12 participants aged between 18 and 55 years with type 1 diabetes for ≥5 years and an HbA_1c_ <75 mmol/mol (9%) were recruited. Individuals were randomised using computer generated block randomisation to start with one episode of HIT (4 × 30 s cycle sprints [2 min recovery] at 150% of maximum wattage achieved during $\dot{V}{\mathrm{O}}_{2\mathrm{peak}}$ assessment) or rest (control). The following day they underwent a 90 min hyperinsulinaemic–hypoglycaemic clamp study at 2.5 mmol/l with measurement of hormonal counterregulatory response, symptom scores and cognitive testing (four-choice reaction time and digit symbol substitution test). Each intervention and subsequent clamp study was separated by at least 2 weeks. The participants and investigators were not blinded to the intervention or measurements during the study. The investigators were blinded to the primary outcome and blood analysis results.

**Results:**

All participants (six male and six female, age 19–54 years, median [IQR] duration of type 1 diabetes 24.5 [17.3–29.0] years, mean [SEM] HbA_1c_ 56 [3.67] mmol/mol; 7.3% [0.34%]) completed the study (both interventions and two clamps). In comparison with the rest study, a single episode of HIT led to a 29% increase in the adrenaline (epinephrine) response (mean [SEM]) (2286.5 [343.1] vs 2953.8 [384.9] pmol/l); a significant increase in total symptom scores (Edinburgh Hypoglycaemia Symptom Scale: 24.25 [2.960 vs 27.5 [3.9]; *p*<0.05), and a significant prolongation of four-choice reaction time (591.8 [22.5] vs 659.9 [39.86] ms; *p*<0.01] during equivalent hypoglycaemia induced the following day.

**Conclusions/interpretation:**

These findings are consistent with the hypothesis that IAH develops in people with type 1 diabetes as a habituated response and that introduction of a novel stressor can restore, at least partially, the adapted counterregulatory hormonal, symptomatic and cognitive responses to hypoglycaemia.

**Trial registration:**

ISRCTN15236211.

**Electronic supplementary material:**

The online version of this article (10.1007/s00125-019-05076-5) contains peer-reviewed but unedited supplementary material, which is available to authorised users.

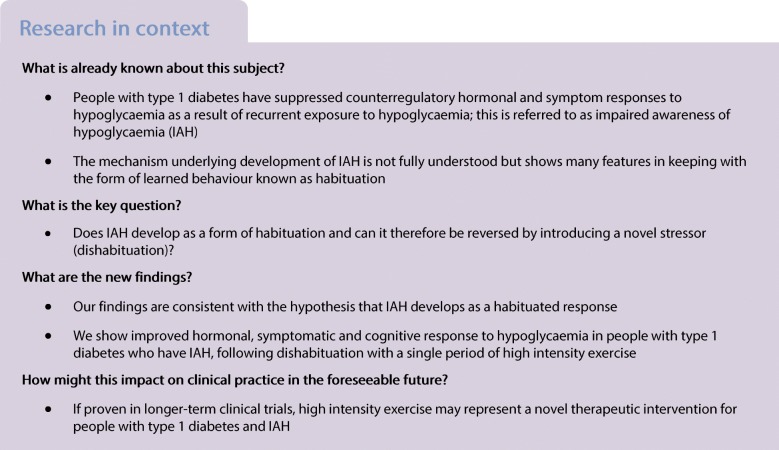



## Introduction

Severe hypoglycaemia is an adverse effect of insulin therapy in diabetes that has a well-recognised morbidity and mortality [1]. Fear of hypoglycaemia often outweighs concerns about long-term consequences of chronic hyperglycaemia [2], and acts as a barrier to patients achieving optimal glycaemic control [3]. The major risk factor for severe hypoglycaemia is impaired awareness of hypoglycaemia (IAH), which increases the risk of an event six-fold [4]. People with IAH have a reduced ability to perceive the onset of acute hypoglycaemia [5]. It affects 25% of all people with type 1 diabetes and, of concern, the incidence of IAH has not changed in the last 2–3 decades despite the introduction of insulin analogues and improved insulin delivery systems [6]. IAH is an acquired abnormality that should be placed alongside chronic microvascular complications such as retinopathy, neuropathy and nephropathy as it can be just as serious and disabling.

The average individual with type 1 diabetes will experience about two episodes of symptomatic hypoglycaemia per week [7, 8]. Ordinarily, hypoglycaemia initiates a biological counterregulatory response that comprises a broad range of physiological, symptomatic and behavioural changes that together act to restore normal glucose homeostasis. Failure of this biological response leads to progressive hypoglycaemia and may result in individuals being unable to self-treat or to loss of consciousness (severe hypoglycaemia). In their seminal study in 1991, Heller and Cryer demonstrated that prior exposure to hypoglycaemia leads to suppression of both hormonal and symptomatic responses during hypoglycaemia of the same depth and duration induced the following day [9]. This finding was later confirmed in people with type 1 diabetes [10], and it was further established that increasing the frequency [11] and duration [12] of prior exposure to hypoglycaemia increases the extent of counterregulatory response suppression, while strict avoidance of hypoglycaemia restores counterregulatory responses [13]. Cumulatively, this exposure to recurrent hypoglycaemia leads to the development of IAH where individuals demonstrate a reduced magnitude and increased threshold (lower glucose) for sympathoadrenal activation and symptomatic experience of hypoglycaemia. Unfortunately, despite nearly three decades since Heller’s and Cryer’s seminal work, the biological mechanisms underpinning the development of IAH remain unknown.

Habituation is a form of adaptive memory that develops in many organisms in response to a repeated, often stressful stimulus [14]. Habituation has been defined as a reduction in the psychological, behavioural or physiological response to a stimulus as a result of recurrent or prolonged exposure [14, 15]. Individuals with type 1 diabetes who develop IAH demonstrate reduced physiological (hormonal and symptomatic), psychological (reduced anxiety) and behavioural (inappropriate responses to hypoglycaemia while driving) responses to hypoglycaemia [14–18]. This led us to propose that IAH may represent a habituated response to repeated hypoglycaemia. To test this hypothesis, we recently examined in a rodent model whether IAH could be reversed, at least temporarily, by the introduction of a single novel stress stimulus, a phenomenon referred to as dishabituation. Rodents were exposed to non-severe hypoglycaemia three times a week for a period of 4 weeks (habituation), and subsequently randomised to a single burst of high intensity training (HIT; dishabituation) or control (no exercise) before undergoing a controlled experimental hypoglycaemia study 24 h later [19]. In comparison with control animals, those who had been exposed to the dishabituatory stimulus had a significantly greater counterregulatory hormonal response during the controlled hypoglycaemia study [19]. While this finding supported the hypothesis that IAH develops as a result of habituation, the rodents did not have type 1 diabetes and the counterregulatory response was assessed via hormonal measures only [19]. In the current proof-of-concept study, we sought to further test the habituation hypothesis by examining whether a single burst of HIT would act as a dishabituatory stimulus in people with long-duration type 1 diabetes who had IAH.

## Methods

This was a single-centre, randomised crossover study carried out at Ninewells Hospital, Dundee, Scotland. Ethics approval was obtained from an independent research ethics committee (17/SS/0150) and the study was registered with the International Standard Randomised Controlled Trials Register (ISRCTN15236211). The study was conducted in accordance with the Declaration of Helsinki, and written informed consent was obtained from all participants before inclusion in the study.

Inclusion criteria were: diagnosis of type 1 diabetes for at least 5 years, treatment with multiple daily injections or continuous subcutaneous insulin infusion, HbA_1c_ <75 mmol/mol (9%), age of 18 to 55 years, and IAH (Gold score [4] ≥4, modified Clarke score [20] ≥4 or Dose Adjustment For Normal Eating [DAFNE] hypoglycaemia awareness rating [21] 2 or 3). Exclusion criteria were: history of significant cardiac, renal, respiratory or neurological disease, pregnancy or breastfeeding, treatment with beta-blockers, history of high-risk foot disease, competitive sportsman [22] or participation in HIT in the preceding 6 months, or physical disability that might limit exercise.

The randomisation relates to the order that the HIT or rest intervention was carried out by the participant. Randomisation was carried out in a good clinical practice (GCP)-compliant manner using http://www.randomization.com/.

Participants were identified using the Scottish Diabetes Research Network (SDRN) and from the diabetes outpatient clinics in NHS Tayside, Scotland. The study took place at the Clinical Research Centre, Ninewells Hospital and Medical School, Dundee.

**Fig. 1:**
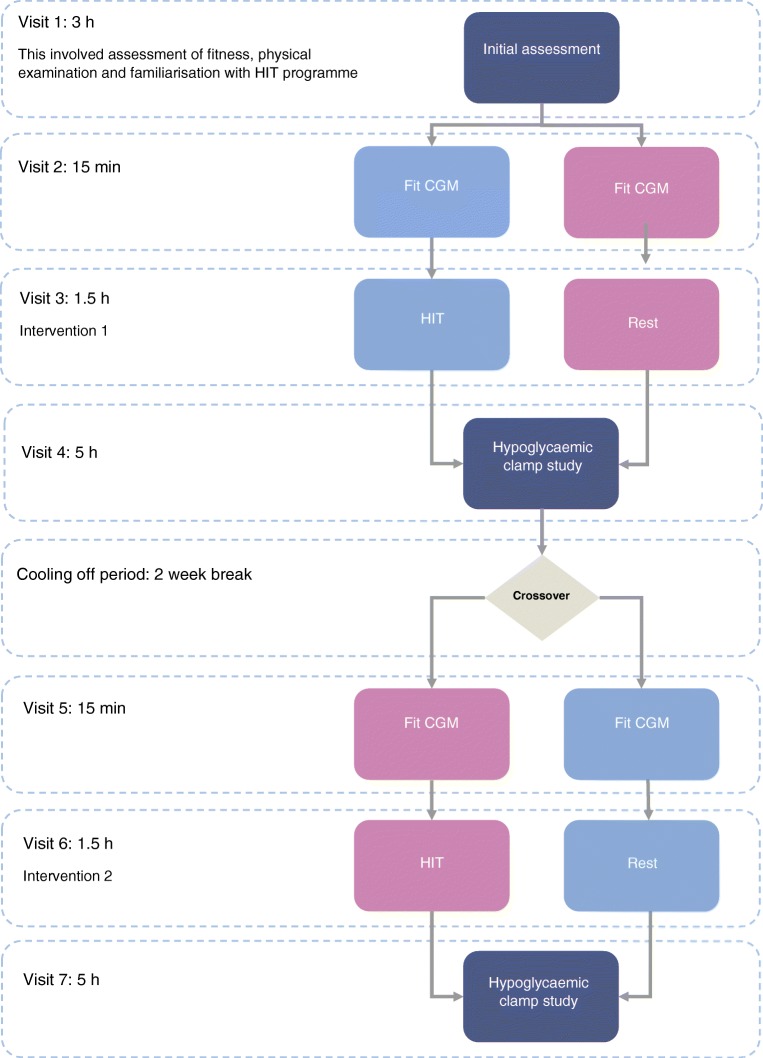
Study flow diagram showing the different stages of the study

After obtaining informed written consent and initial screening, which included confirmation of IAH, each participant attended the Clinical Research Centre on seven separate occasions (See Fig. 1). During visit 1 anthropometric measurements, physical examination and electrocardiogram were performed. In addition, participants undertook an incremental maximal to volitional exhaustion exercise test using on a cycle ergometer (Corival, Lode, Groningen, the Netherlands; Metamax 3B, CORTEX Biophysik, Leipzig, Germany) to determine their $\dot{V}{\mathrm{O}}_2$ and peak heart rate.

For this study we have defined HIT as episodic short bursts of high intensity exercise separated by short periods of recovery at lower intensity [23]. The HIT programme used in this study was 20 min in duration; 5 min of gentle warm up cycling at 50 W, 40–60 rev/min followed by 4 × 30 s cycle sprints [2 min recovery] at 150% of maximum wattage achieved during $\dot{V}{\mathrm{O}}_{2\mathrm{peak}}$ assessment with an aim to achieve ≥90% peak heart rate; concluding with 5 min cool down at 50 W, 40–60 rev/min. Standardised advice [24] was given regarding blood glucose levels and insulin dose adjustment prior to and following HIT. The control intervention was 20 min of seated rest. Venous blood samples were taken for counterregulatory hormones (adrenaline, noradrenaline [norepinephrine], glucagon and lactate) at baseline of both the rest and HIT intervention visits with samples also taken after the third and fourth bouts of exercise during the HIT intervention. Each participant underwent a single HIT programme or controlled rest intervention the afternoon before undergoing a hyperinsulinaemic–hypoglycaemic clamp study the following day. The hyperinsulinaemic–hypoglycaemic clamp study was used to assess their hormonal counterregulatory response, symptom awareness and cognitive response. Hyperinsulinaemic–hypoglycaemic clamp study is considered the gold standard for comparing counterregulatory response to equivalent hypoglycaemia [25]. There was a 2 week cooling off period between the interventions to avoid any lasting effect prior to crossing in to the second arm of the study.

### Experimental hypoglycaemia

For each of the two clamp study visits (separated by at least 2 weeks) participants initially attended to be fitted with a real-time continuous glucose monitor (CGM) (Dexcom G4, Dexcom, San Diego, CA, USA), with low glucose alarm (set at 4.0 mmol/l), at least 48 h before the study, to ensure absence of significant hypoglycaemia (<3 mmol/l for >20 min) [26] before the clamp procedure.

The evening preceding the clamp study, participants were advised to reduce their night-time basal insulin by ≈20% and fast for at least 8 h before coming to the Clinical Research centre at 08:00 hours. On the morning of the clamp, a retrograde cannula was inserted into a dorsal vein of the non-dominant hand. It was then placed in a heated box at 55–60°C to arterialise venous blood. This line was used for blood sampling during the clamp study. In the contralateral arm, the antecubital vein was cannulated and used for insulin and dextrose infusions.

Prior to commencing the hypoglycaemic clamp, CGM data were analysed to ensure there was no hypoglycaemia in the preceding 24 h. If there was evidence of hypoglycaemia the study was postponed and the intervention visit repeated. The study was postponed and intervention repeated three times in total by two participants: one participant had to repeat both interventions and one had to repeat the rest intervention alone.

The insulin infusion (0.3 U/ml) was started at a rate of 50 ml/h for priming purposes until the blood glucose dropped to below 7 mmol/l into the euglycaemic range, after which a rate of 1.5 mU^−1^ kg^−1^ min^−1^ was maintained for the duration of the clamp. 20% dextrose was simultaneously infused at a variable rate (Infusomat Space, B. Braun Medical, Sheffield, UK) based on frequent (every 5 min) bedside plasma glucose measurements (Biosen C-Line GP+, EKF diagnostics, Cardiff, UK) to maintain blood glucose at pre-determined levels. Euglycaemia (glucose 4–6 mmol/l) was achieved and maintained for the first 30 min of the glucose clamp study, and subsequently, blood glucose was reduced over 30 min to a final glucose level of 2.5 mmol/l. Blood glucose was maintained at 2.5 mmol/l for 60 min before glucose levels were returned to the euglycaemic range.

### Counterregulatory hormones

Arterialised blood for insulin and counterregulatory hormones (adrenaline, noradrenaline, glucagon and lactate) was taken every 30 min (*t* = −30, 0, 30, 60 and 90 min) during the clamp.

### Symptoms

At each 30 min time point, participants completed a validated questionnaire, the Edinburgh Hypoglycaemia Symptom Scale (EHS) [27], scoring 11 symptoms from 1 (not at all) to 7 (very severe) on a visual analogue scale.

### Cognitive function tests

A series of psychometric tests recognised to be sensitive to hypoglycaemia were carried out in the same order, starting approximately 2 min before each 30 min time point: digit symbol substitution test (DSST) [28] and four-choice reaction time (4CRT) [29].

### Laboratory assays

Arterialised blood samples were taken every 5 min during the clamp and centrifuged, and plasma glucose was analysed at the bedside by an enzymatic–amperometric method using chip-sensor technology (Biosen C-Line GP+). Samples taken at 30 min intervals were centrifuged within 1 h to separate the serum or plasma and stored at −80°C before assay. Hormone levels of insulin (Alpco, Salem, NH, USA; CV inter 4.9%, intra 7.2%), adrenaline (Alpco; CV inter 19.6%, intra 14.3%), noradrenaline (Alpco; CV inter 16.3%, intra 12.0%), glucagon (EMD Millipore, Billerica, MA, USA; CV inter 9.8%, intra 6.52%) and lactate (Siemens Advia 2100, Siemens Healthcare Diagnostics, Tarrytown, NY, USA) were measured, and samples were analysed in duplicate according to the manufacturer’s instructions.

### Data and statistical analysis

The predefined primary endpoint was the difference in adrenaline response at a glucose level of 2.5 mmol/l following rest and HIT. Power calculations were based on a previous hypoglycaemic clamp study performed in a similar cohort of participants with type 1 diabetes and IAH conducted in our laboratory [30]. For a matched analysis, ten participants were required to detect a difference of 1 SD with α set at 0.05 and 80% power. Twelve participants were recruited to allow for any participants who did not complete both experimental studies. Secondary outcomes examined were: difference in symptom awareness score, cognitive function and other counterregulatory hormone responses during hypoglycaemia (2.5 mmol/l) following rest and HIT intervention. For the primary and secondary endpoints, a generalised estimated equation was used adjusting for order effect and baseline as covariates. A *p* value <0.05 was considered statistically significant. Paired *t* tests were used to assess hormonal and heart rate response to HIT. All data are presented as mean ± SEM. Statistical analyses were conducted using IBM SPSS Statistics 22 software, IBM, Armonk, NY, USA.

## Results

### Participant characteristics

**Fig. 2:**
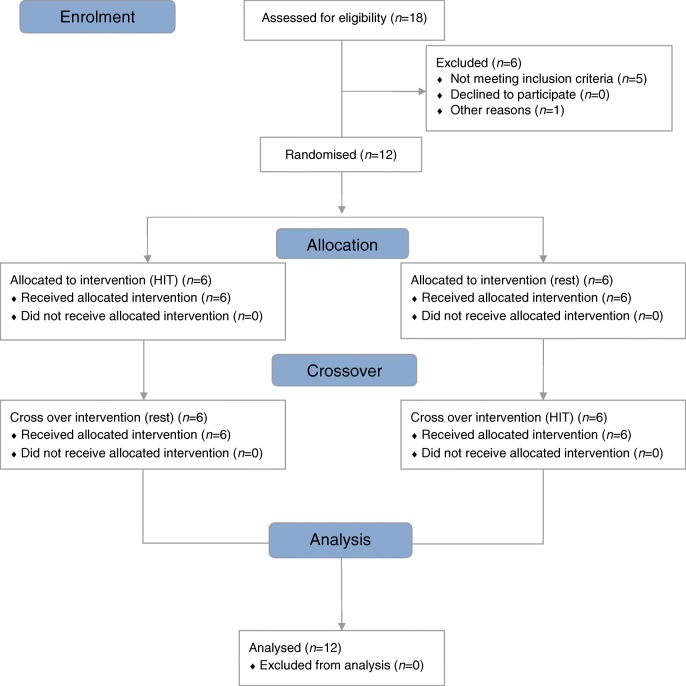
Consort diagram showing participant allocation at each stage of the study

Recruitment ran from January 2018 to August 2018. Of the 18 participants screened, five did not meet the inclusion criteria and one was withdrawn; 12 participants gave informed written consent and were randomised (see Fig. 2). The 12 participants (six male and six female, aged 19–54 years) who completed the study had long-duration type 1 diabetes (median [IQR] duration of type 1 diabetes 24.5 [17.3–29.0] years] and IAH defined by at least one of a Gold score ≥4 (7/12 participants), modified Clarke] score ≥4 (10/12 participants) or DAFNE hypoglycaemia awareness rating 2 or 3 (11/12 participants) (electronic supplementary material [ESM] Table 1). Mean (SEM) HbA_1c_ at randomisation was 56 (3.67) mmol/mol; 7.3% (0.34).

### Physiological response to HIT in type 1 diabetes with IAH

**Fig. 3:**
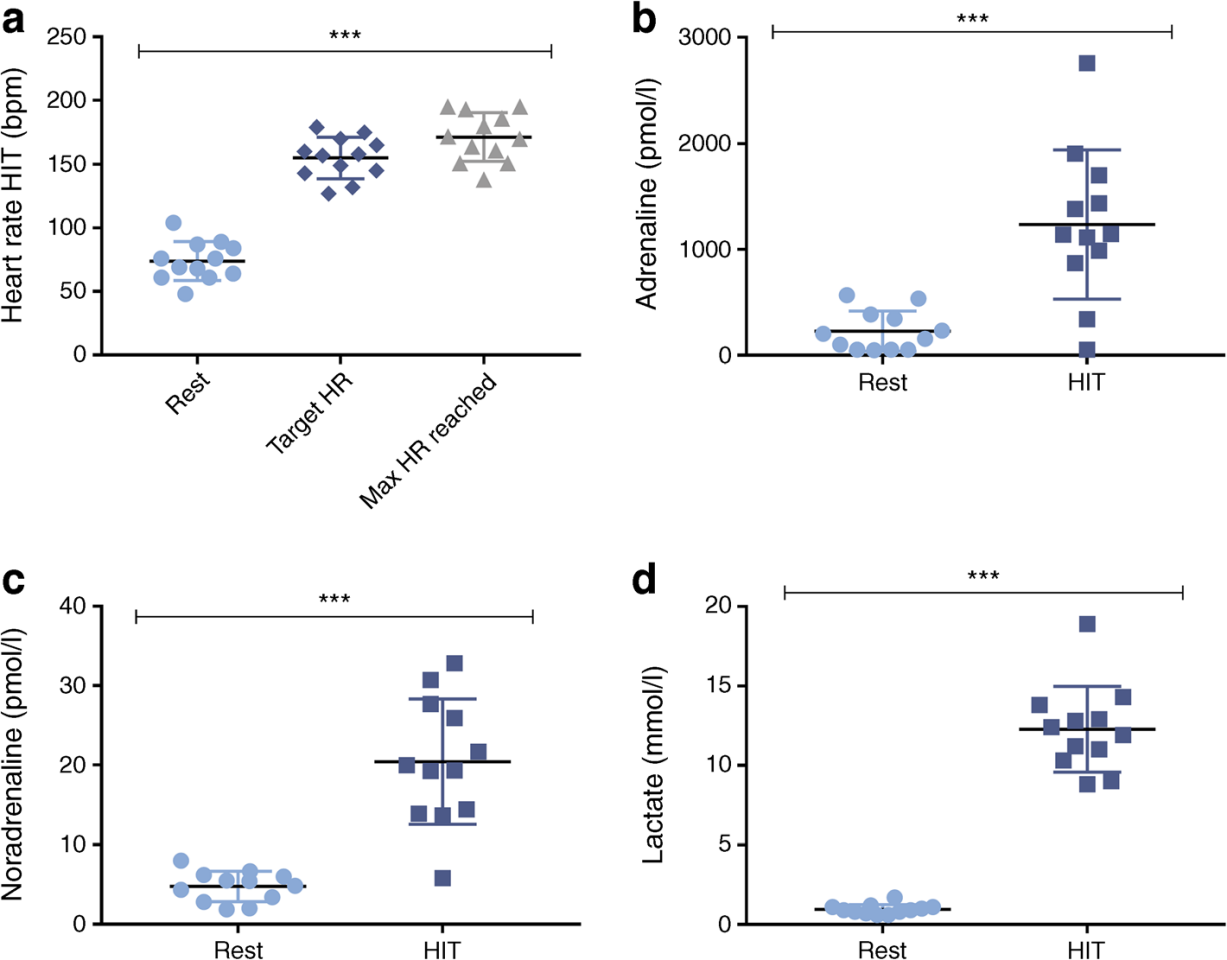
Physiological responses to HIT for each participant. (**a**) Heart rate during rest, at the 90% maximum heart rate target determined from the $\dot{V}{\mathrm{O}}_{2\mathrm{peak}}$test, and the maximum heart rate reached during HIT. (**b**, **c**, **d**) Adrenaline (**b**), noradrenaline (**c**) and lactate (**d**) during rest and HIT interventions. *n* = 12; values are the mean ± SEM. ***p< 0.001 by paired *t* test comparing rest with HIT or max HR reached. HR, heart rate; max, maximum

All participants were able to complete the HIT intervention which involved 4 × 30 s cycle sprints (2 min recovery) at 150% of maximum wattage achieved during $\dot{V}{\mathrm{O}}_{2\mathrm{peak}}$ assessment. Each participant achieved their target >90% peak heart rate (Fig. 3a). The HIT programme provided an intense stimulus to adrenaline (mean [SEM] 228.45 [54.65] vs 1236.83 [203.42] pmol/l; rest vs HIT, *p*<0.001] (Fig. 3b) and noradrenaline (4.77 [0.55] vs 20.42 [2.28] pmol/l; *p*<0.001] (Fig. 3c), and resulted in a marked 12-fold increase in lactate (0.95 [0.09] vs 12.28 [0.78] mmol/l; *p*<0.001) (Fig. 3d).

### Dishabituation with HIT in type 1 diabetes with IAH

**Fig. 4:**
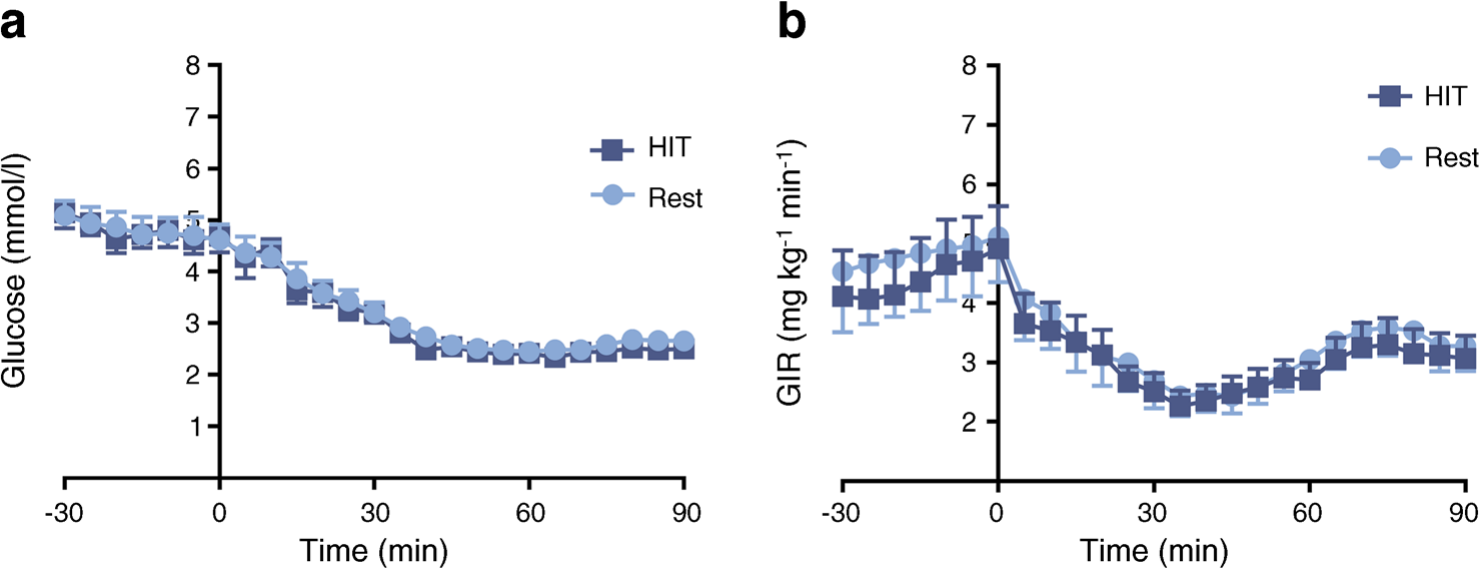
Hyperinsulinaemic–hypoglycaemic clamp profiles following HIT and rest (control). (**a**) Plasma glucose level. (**b**) Glucose infusion rate. *n* = 12; values are the mean ± SEM. GIR, glucose infusion rate

The day following HIT or rest interventions, participants underwent a hyperinsulinaemic–hypoglycaemic clamp to assess their counterregulatory response. During the clamp, plasma glucose profiles were well matched (Fig. 4a) at baseline (mean [SEM] 4.81 [0.06] vs 4.80 [0.07] mmol/l [rest vs HIT]) and at steady state hypoglycaemia (30–90 min) (2.65 [0.06] vs 2.54 [0.06] mmol/l; *p* = 0.23). Mean glucose infusion rates (30-90 min) were lower following HIT (2.80 [0.10] vs 3.07 [0.12]), but this difference was not significant; *p* = 0.23 (Fig. 4b). Plasma insulin was maintained at 704.1 (17.2) vs 655.3 (17.2) pmol/l throughout the clamp period (*p* = 0.30).

### Hormonal counterregulatory response

**Fig. 5:**
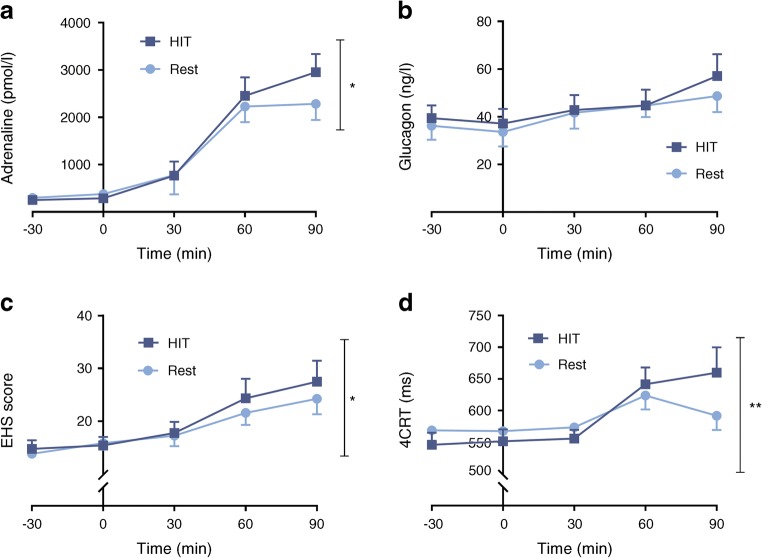
Acute exposure to HIT improves defective counterregulatory response. Plasma adrenaline (**a**), glucagon (**b**), total EHS score (**c**) and 4CRT (**d**) during the hyperinsulinaemic–hypoglycaemic clamp study. *n* = 12; values are the mean ± SEM. *p<0.05, **p<0.01 using the generalised estimate equation

The single HIT intervention had an overall significant effect on the adrenaline response to subsequent hypoglycaemia. Mean (SEM) 90 min hypoglycaemia-induced adrenaline following rest and HIT interventions, respectively, was 2286.5 (343.1) vs 2953.8 (384.9) pmol/l; *p*<0.05 (Fig. 5a). A smaller, non-significant rise in noradrenaline (5308.3 [656.5] vs 5754.8 [771.2] pmol/l) also followed HIT (*p* = 0.196). Interestingly, although not of statistical significance, there was a small increase in glucagon during equivalent hypoglycaemia following HIT (48.7 [6.7] vs 57.1 [9.1] ng/l; *p* = 0.238; Fig. 5b).

### Symptomatic response

Following HIT total symptom scores (where the minimal possible score is 11) measured during subsequent hypoglycaemia were significantly increased (Mean [SEM] EHS score: 24.25 [2.96] vs 27.5 [3.9], respectively, rest vs HIT, *p*<0.05; Fig. 5c). However, of the EHS subcategories, while there was a numerically greater autonomic symptom score (11.75 [1.50] vs 12.83 [1.74], rest vs HIT, respectively, *p* = 0.144] and neuroglycopenic symptom score (9.75 [1.46] vs 11.42 [2.11], respectively, *p* = 0.174) in response to hypoglycaemia, these did not achieve significance.

### Cognitive response

We found that the 4CRT during hypoglycaemia was significantly prolonged following HIT (mean [SEM] 591.8 [22.5] vs 659.9 [39.9] ms; rest vs HIT, respectively, *p*<0.01; Fig. 5d). There was no difference in the DSST during hypoglycaemia following rest or HIT.

## Discussion

Habituation is a form of adaptive learning where the response to a stimulus is reduced or ceases altogether following repeated exposure to the stimulus. This is often considered in the context of innate behaviours [14, 15]. The gill-withdrawal (sensory–motor) reflex in *Aplysia* [31] is a classic model of habituation. When the gill-withdrawal reflex is repeatedly induced by a tactile external stimulus to the siphon, the scale of the response is markedly diminished (habituation). Hypoglycaemia can be considered an internal sensory stimulus, which leads in turn to a reflex counterregulatory (motor) response [32]. If organisms adapt to repeated hypoglycaemia through habituation then it would be predicted that repeated exposure to hypoglycaemia would lead to a markedly diminished response. This is indeed the case as has been shown in humans [9, 10] and rodents [33], and as with a habituated response, we see widespread and progressive suppression of physiological (e.g. suppressed counterregulatory hormonal and symptomatic responses), behavioural (e.g. reduced drive to feed, impairment when driving [17]) and psychological (e.g. reduced anxiety) responses to hypoglycaemia as a result of repeated exposure [16]. Furthermore, a cardinal feature of the habituated gill-withdrawal reflex in *Aplysia* is that it can then be restored, at least temporarily, by applying a strong tactile stimulus to another part of the animal (dishabituation) [31]. Our present findings, supported by our previous work in rodents [19], that hormonal, symptom and cognitive responses to hypoglycaemia can be at least partially restored following a single episode of HIT in individuals with long-standing type 1 diabetes who have been exposed to multiple episodes of hypoglycaemia, therefore provides robust support for the hypothesis that IAH develops through habituation to recurrent hypoglycaemia.

There are nine proposed criteria that define habituation [15]. As described, criterion 1: ‘Given that a particular stimulus elicits a response, repeated applications of that stimulus result in a decreased response (habituation)’ [15], is characteristic of recurrent hypoglycaemia [9, 10]. Similarly, criterion 2: ‘If the stimulus is withheld, the response tends to recover over time (spontaneous recovery)’ [15], and we know that hypoglycaemia avoidance can lead to recovery of counterregulatory responses to subsequent hypoglycaemia [13, 34]. In this study, we have addressed criterion 8: ‘Presentation of another (usually strong) stimulus results in recovery of the habituated response (dishabituation)’, and have shown that it is possible, at least temporarily, to recover hypoglycaemia counterregulation. Interestingly, criterion 7: ‘Habituation of response to a given stimulus exhibits stimulus generalization to other stimuli’, may explain why our results differ from previous work suggesting that exercise can further suppress counterregulatory responses to hypoglycaemia [35, 36]. It is possible that the mild–moderate exercise regimen, leads through stimulus ‘generalisation’, to suppression of the subsequent counterregulatory response to insulin-induced hypoglycaemia. The key differentiating feature between the studies being the intensity of the different stimulus.

Habituation is considered an adaptive survival response to repeated exposure of a physiological stressor in order to develop a degree of tolerance of that specific stressor. In contrast, the physiological response to repeated hypoglycaemia is currently considered maladaptive and is often referred to as hypoglycaemia-associated autonomic failure (HAAF), as originally proposed by Cryer [37]. The term HAAF is, however, misleading because, as mentioned, the suppressed counterregulatory response can be reversed in many people through strict avoidance of hypoglycaemia [13, 34]. This means the autonomic system does not ‘fail’, and instead is more likely to be adapting to the presence or absence of repeated hypoglycaemia. Pre-clinical studies are supportive of the habituation hypothesis in that recurrent hypoglycaemia triggers a series of changes at a cellular level that act to induce tolerance, facilitating organisms to cope better when subsequently deprived of energy. For example, prior recurrent hypoglycaemia protects against neuronal death during subsequent very severe hypoglycaemia [38]. Glucocorticoids [39] and urocortin [40], which are key to neuroprotection and initiation of adaptive cellular mechanisms [41], are also integral to the organism’s response to recurrent hypoglycaemia. Therefore, these systematic and cellular responses to recurrent hypoglycaemia also point to IAH developing as an adaptive, habituated response to repeated hypoglycaemic stress.

In the current study we employed HIT as the novel dishabituating stimulus. We confirmed the effectiveness of the HIT intervention as a novel stimulus by demonstrating that it evoked a marked rise in heart rate, adrenaline and lactate. The day following the HIT (or rest) intervention, participants underwent a controlled hypoglycaemia study where we demonstrated significant increases induced by preceding HIT in the hormonal counterregulatory response. There was also, and of relevance in clinical practice, an increase in total symptom scores (EHS [27]) in response to matched hypoglycaemia following HIT, indicating participants were more aware of hypoglycaemia. Finally, the 4CRT was prolonged during hypoglycaemia following HIT. Choice reaction time is used to examine psychomotor and attentional focus. Acute hypoglycaemia markedly impairs performance in almost all aspects of executive function in adults with type 1 diabetes, with time taken to complete cognitive testing taking considerably longer [42, 43]. Zammitt et al have shown that 4CRT is also prolonged during experimental hypoglycaemia in people with type 1 diabetes and intact awareness, but not in those with IAH [44]. Our finding of a significant slowing of 4CRT during hypoglycaemia in participants with IAH following HIT is therefore indicative that dishabituation has, at least partially, restored counterregulatory hormonal, symptom and cognitive responses towards that seen in individuals with intact awareness.

There are a number of limitations to our study. Although the hyperinsulinaemic–hypoglycaemic clamp is the gold standard technique for assessing counterregulatory responses to hypoglycaemia [25], the studies are conducted under strictly controlled conditions in a laboratory and, as such, the experience of hypoglycaemia may not fully reflect its real-world experience. We also used three different hypoglycaemia awareness questionnaires [4, 20, 21], which were not concordant for all participants. However, 11/12 participants scored 2 or 3 on the DAFNE hypoglycaemia awareness rating and these individuals show a fourfold increased risk of severe hypoglycaemia [21], while 10/12 participants had a modified Clarke score ≥4, which carries a 4.6-fold increased risk of severe hypoglycaemia [45].

Overall, the current study supports the hypothesis that IAH in type 1 diabetes develops as a result of habituation. This is not firmly established as additional studies will need to be performed to test the other features of habituation. However, consistent with our hypothesis, we have demonstrated that the introduction of HIT as a dishabituating stimulus, in people with long-standing type 1 diabetes and IAH, acutely restores a broad array of physiological, symptomatic and cognitive responses during subsequent experimental hypoglycaemia. If this effect is maintained following a more prolonged intervention with HIT, it may offer a novel, therapeutically attractive strategy to improve and restore hypoglycaemia awareness in people with type 1 diabetes and reduce the risk of severe hypoglycaemia.

## Electronic supplementary material

ESM Table 1 (PDF 64 kb)

## Data Availability

Data are available on request from the corresponding author.

## Abbreviations

4CRT: Four-choice reaction time
CGM: Continuous glucose monitor
DAFNE: Dose Adjustment For Normal Eating
DSST: Digit symbol substitution test
EHS: Edinburgh Hypoglycaemia Symptom Scale
HAAF: Hypoglycaemia-associated autonomic failure
HIT: High intensity training
IAH: Impaired awareness of hypoglycaemia
