# Supplementary material for: A randomised controlled study of high intensity exercise as a dishabituating stimulus to improve hypoglycaemia awareness in people with type 1 diabetes: a proof-of-concept study
Source: Diabetologia. 2020 Jan 15;63(4):853–63. doi: 10.1007/s00125-019-05076-5 (PMC7054230; doi:10.1007/s00125-019-05076-5)
Supplement: Supplementary file 1 — (PDF 64 kb) [file 125_2019_5076_MOESM1_ESM.pdf]

Participant number	IAH		
	Gold	Clarke	DAFNE
1	3	3	2
2	4	4	1
3	3	5	2
4	4	7	2
5	3	4	2
6	6	4	2
7	3	5	2
8	5	8	3
9	7	5	2
10	4	6	2
11	4	5	2
12	1	3	2
Mean	4	5	2

ESM Table 1: Validation of hypoglycaemia awareness; Gold, Clarke and DAFNE questionnaire results
